# GWAS Procedures for Gene Mapping in Diverse Populations With Complex Structures

**DOI:** 10.21769/BioProtoc.5284

**Published:** 2025-04-20

**Authors:** Zhen Zuo, Mingliang Li, Defu Liu, Qi Li, Bin Huang, Guanshi Ye, Jiabo Wang, You Tang, Zhiwu Zhang

**Affiliations:** 1 Electrical and Information Engineering College, Jilin Agricultural Science and Technology University, Jilin, Jilin, China; 2 Information Technology Academy, Jilin Agricultural University, Changchun, Jilin, China; 3 Key Laboratory of Qinghai-Tibetan Plateau Animal Genetic Resource Reservation and Utilization, Sichuan Province and Ministry of Education, Southwest Minzu University, Chengdu, Sichuan, China; 4 Department of Crop and Soil Sciences, Washington State University, Pullman, WA, USA

**Keywords:** Statistical power, False discovery, Complex trait, Candidate gene, Principal component

## Abstract

With reduced genotyping costs, genome-wide association studies (GWAS) face more challenges in diverse populations with complex structures to map genes of interest. The complex structure demands sophisticated statistical models, and increased marker density and population size require efficient computing tools. Many statistical models and computing tools have been developed with varied properties in statistical power, computing efficiency, and user-friendly accessibility. Some statistical models were developed with dedicated computing tools, such as efficient mixed model analysis (EMMA), multiple loci mixed model (MLMM), fixed and random model circulating probability unification (FarmCPU), and Bayesian-information and linkage-disequilibrium iteratively nested keyway (BLINK). However, there are computing tools (e.g., GAPIT) that implement multiple statistical models, retain a constant user interface, and maintain enhancement on input data and result interpretation. In this study, we developed a protocol utilizing a minimal set of software tools (BEAGLE, BLINK, and GAPIT) to perform a variety of analyses including file format conversion, missing genotype imputation, GWAS, and interpretation of input data and outcome results. We demonstrated the protocol by reanalyzing data from the Rice 3000 Genomes Project and highlighting advancements in GWAS model development.

## Graphical overview


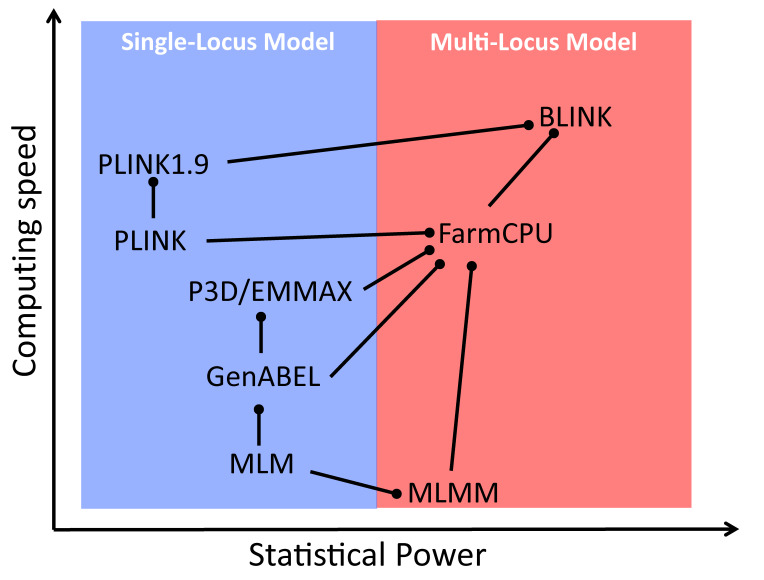


**Comparison of GWAS models for statistical power and computing speed.** The models are connected by lines with circle heads when direct comparisons exist in the literature. The models marked with circle heads have better power (vertical) or speed (horizontal). When power and speed are in contradiction, power is used. MLM: mixed linear model; CMLM: compressed MLM; ECMLM: enriched CMLM; SUPER: settlement of MLM under progressively exclusive relationship; FarmCPU: fixed and random model circulating probability unification; BLINK: Bayesian-information and linkage-disequilibrium iteratively nested keyway.

## Background

The genome-wide association study (GWAS) is currently the primary method for mapping genes of interest and identifying genetic markers to predict the risk of human diseases and conduct marker-assisted selection for molecular breeding in animals and plants. Introduced in human genetic studies at the end of the last century, GWAS is a statistical inference of linkage disequilibrium (LD; please see Table S1 for all abbreviation definitions) caused by genetic linkage and many other factors, such as mutation, selection, and non-random mating [1–3]. Since GWAS aims to map genes based primarily on genetic linkage, excluding the influence of remaining factors during experimental design and data analysis is crucial [4,5].

Gene mapping depends on linkage analysis through crossing, especially in plants. As crossing is conducted in recent generations under controlled experiments, linkage analysis only needs to address type I errors. The corresponding drawback is the low mapping resolution due to limited recombination events over a small number of generations [6,7]. In contrast, GWAS relies on historical recombinations accumulated over many generations, resulting in high-resolution mapping at the gene level. Because genetic linkage is not the only reason for LD accumulated historically, GWAS must address both type I errors and spurious associations arising from population structure and selection [8–10].

With increasing marker density and complexity of enlarged plant samples, researchers face four roadblocks toward reporting their discoveries. The first roadblock is computing hardware and time. A genotype file of 100 GB is problematic for a computer with only 64 GB memory to analyze using R software packages without reading from the disk. For metabolic data, gene expression data, and electronic data with thousands of traits, computing time becomes a concern when analyzing a single trait takes several hours [11].

The second roadblock is statistical power, especially for small samples and traits with low heritability [12,13]. Although there are many statistical models and computing software packages with varied statistical power, researchers are not familiar with the majority and only use the ones they are familiar with, especially the ones developed earlier. Researchers gain confidence by seeing the overlaps with enlarged datasets using familiar statistical models and software, especially with published results, which is a cause of chain of false discoveries [14–16].

The third roadblock is to conduct GWAS properly. There are many factors that invite false positives, including phenotypic outliers and rare alleles of genetic markers in small samples [17]. Any combination of the three factors (outliers, rare allele, and small sample) could lead to false positives. The combination of outliers and rare alleles also leads to false positives even in large samples.

The fourth roadblock is to properly interpret phenotypic data, genotypic data, and association results. The most common mistake is inappropriately setting experiment-wise thresholds, leading to either hidden or false discoveries [18–20]. For simplicity, many researchers choose Bonferroni multiple test correction [21]. This threshold is over-conservative, especially for GWAS with dense markers where many markers are in close genetic linkage. The number of independent tests is far lower than the number of markers [22,23]. The opposite mistake is to underestimate the number of independent tests experiment-wise. When hundreds or thousands of traits are analyzed, a threshold of a single test type I error (e.g., 1%) divided by the number of independent markers generates far more false positives than expected from the single test type I error. The number of traits should be part of multipliers for the multiple test correction [24].

Our objective is to address these roadblocks so that researchers can conduct GWAS efficiently and produce truthful discoveries. First, we will provide an overview of statistical models and computing software to help readers decide whether to continue using familiar tools or explore advanced alternatives. Second, we will provide a step-by-step protocol showing how to properly conduct GWAS. Lastly, we will showcase a real publicly available rice dataset and demonstrate how to conduct GWAS and interpret phenotypic and genotypic data and analytical results. To allow readers to easily replicate our results and apply them to their own data, we limited our computing tools to only three software packages (BEAGLE [25], BLINK [26], and GAPIT [16,27,28]) for missing genotype imputation, GWAS, data management, analyses, and reports with publication-ready tables and figures.

## Software and datasets

**Model and software selection**

Population structure is one of the most important causes of spurious association. Population structure matrix [4] or principal components [29] were incorporated as covariates to reduce spurious associations using general linear models (GLM) as implemented in PLINK [14], TASSEL [15], and GenABEL [30]. These software packages are intensively used due to computationally efficient GLM and popularity (Table 1). Multiple functions, such as file format conversion and linkage disequilibrium analysis, also contribute to their popularity [14,15].

**Table 1.** GWAS models and software implementations*

Software	Year	Citations	GLM	MLM	CMLM	ECMLM	SUPER	MLMM	FarmCPU	BLINK
PLINK [14]	2007	28141	√							
TASSEL [15]	2007	5930	√	√	√					
GenABEL [30]	2007	2002	√	√						
EMMA [31]	2008	1783		√						
EMMAx [32]	2010	1991		√						
GCTA [33]#	2011	5811		√						
FaST-LMM [34]	2011	1123		√						
GEMMA [35]	2012	2358		√						
GAPIT [16,27,28]	2012	1676	√	√	√	√	√	√	√	√
MLMM [36]	2012	787						√		
FaST-LMM-Select [34]	2012	351		√			√			
BOLT-LMM [13]	2015	471		√						
FarmCPU [37]	2016	786							√	
BLINK [26]	2019	209								√
rMVP [17]	2021	252	√	√				√	√	

*Sorted by publication year and citations by year as of March 12, 2023. GWAS models include general linear model (GLM), mixed linear model (MLM) [5], compressed MLM (CMLM) [12], enriched CMLM (ECMLM) [38], settlement of MLM under progressively exclusive relationship (SUPER) [39], multiple loci mixed model (MLMM) [36], fixed and random model circulating probability unification (FarmCPU) [37], and Bayesian-information and linkage-disequilibrium iteratively nested keyway (BLINK) [26].

#equivalent to the approach of genomic best linear unbiased prediction (gBLUP) in genomic selection [40–42].

Besides population structure, another addition was introduced to control the unequal relatedness (kinship) among individuals within subpopulations using mixed linear models (MLM) [5]. Individuals’ total genetic effects are fitted as random effects with variance and covariance structure defined by the kinship derived from all genetic markers. Multiple algorithms were developed to improve the computing efficiency of MLM, including EMMA [31], P3D [12] (or EMMAx [32]), FaST-LMM [34], BOLT-LMM [13], and GEMMA [35]. These algorithms have the same statistical power as the regular MLM originally solved by the expectation and maximization algorithm [15]. MLM was also enhanced to improve statistical power by accounting for the confounding factor between testing markers and random individual total genetic effects, which causes false negatives. The models with improved statistical power include compressed MLM (CMLM) [12], enriched CMLM (ECMLM) [38], and FaST-LMM-Select [34] or SUPER [39]. FaST-LMM-Select and SUPER are equivalent. Some of the improved MLM models were implemented in software packages with multiple functions, including TASSEL [15] and GAPIT [16,27,28].

To further subtract the confounding between testing markers and the random individual genetic effects to improve statistical power, associated markers were introduced as covariates in the multiple loci mixed model (MLMM) [36]. The random individual genetic effect and testing markers were placed in a random effect model and in a fixed effect model separately (FarmCPU) [37]. Finally, the random individual genetic effects were completely removed in the BLINK model using GLM only [26]. BLINK uses two GLMs iteratively; one selects the associated markers as covariates with maximization of Bayesian information content, and the other tests markers one at a time with the selected associated markers as covariates. By doing so, BLINK not only retains GLM computing efficiency but also gains statistical power, higher than the other multiple loci models, including FarmCPU (Graphical overview). Some of the multiple loci models were implemented in software packages with multiple functions, including GAPIT [16,27,28] and RMVP [17].

Some of the models and software packages were compared for computation efficiency and statistical power against false discovery rates or type I errors (Graphical overview). GAPIT implemented the most models, from GLM, MLM, to SUPER for the single locus models and from MLMM, FarmCPU, to BLINK for the multiple loci models. The computing speed and statistical power can be examined by less than a handful of lines of R code (Box 1). The first three lines import the GAPIT source code, genotype data, and the genetic map from the GAPIT demo data (http://zzlab.net/GAPIT/data). The data contain 281 maize inbred lines and 3,093 single nucleotide polymorphisms (SNPs). The middle three lines set random seed and simulate phenotypes out of 40 SNPs sampled from the first five chromosomes as quantitative trait nucleotides (QTN) with heritability of 70%. The remaining chromosomes serve as a reference so that false positives can be easily identified. The last line calls GAPIT to conduct GWAS with seven models, including FarmCPU and BLINK. The execution takes approximately five minutes on a standard laptop. Some models consume more time than others. Users can assess the computing times of specific models by examining the result files, e.g., Manhattan plots, in the R working directory.

**Box 1. R code to import GAPIT and genotype data, simulate phenotypes, and conduct GWAS**

**Table:** 

#Import GAPIT and demo data source("http://raw.githubusercontent.com/jiabowang/GAPIT/refs/heads/master/gapit_functions.txt") myGD=read.table(file="http://zzlab.net/GAPIT/data/mdp_numeric.txt",head=T) myGM=read.table(file="http://zzlab.net/GAPIT/data/mdp_SNP_information.txt",head=T) #Simultate QTNs on the first five chromosomes set.seed(99164) index1to5=myGM[,2]<6 mySim=GAPIT.Phenotype.Simulation(GD=myGD[,c(TRUE,index1to5)],GM=myGM[index1to5,], h2=.7, NQTN=40, QTNDist="normal") #GWAS with GAPIT using multiple models myGAPIT=GAPIT(Y=mySim$Y,GD=myGD,GM=myGM,PCA.total=3,QTN.position=mySim$QTN.position, model=c("GLM", "MLM", "CMLM", "SUPER", "MLMM", "FarmCPU", "BLINK"))

GAPIT also combines all the Manhattan plots together for comparison (Figure S1). GLM identifies two QTNs based on the 5% type I error after Bonferroni multiple correction. MLM identifies one, CMLM identifies two, SUPER and MLMM identify seven, FarmCPU identifies eight, and BLINK identifies eleven. Users can replace the demo genotypes and genetic maps with their own, change the number of PCs, specify models, and conduct multiple replicates with different random seeds to achieve a more robust evaluation of the statistical power over different models on specific data sets.

**Real case data**

Rice 3000 genome project data (http://ricediversity.org/data/index.cfm) were used for real case analysis. This dataset includes 700K SNPs and 847 accessions published with the GWAS results of rice grain length [43]. The genotype data in PLINK format can be downloaded from http://ricediversity.org/data/sets_hydra/HDRA-G6-4-RDP1-RDP2-NIAS-2.tar.gz. The map file can be downloaded from http://ricediversity.org/data/sets_hydra/HDRA-G6-4-SNP-MAP.tar.gz. The phenotype data file can be downloaded from http://ricediversity.org/data/sets_hydra/phenoAvLen_G6_4_RDP12_ALL.tar.gz. The map version of genotype data is Rice Genome Project version 7. The genome position of known genes was searched from the phytozome database (https://phytozome-next.jgi.doe.gov/).

## Procedure

**Figure 1.**
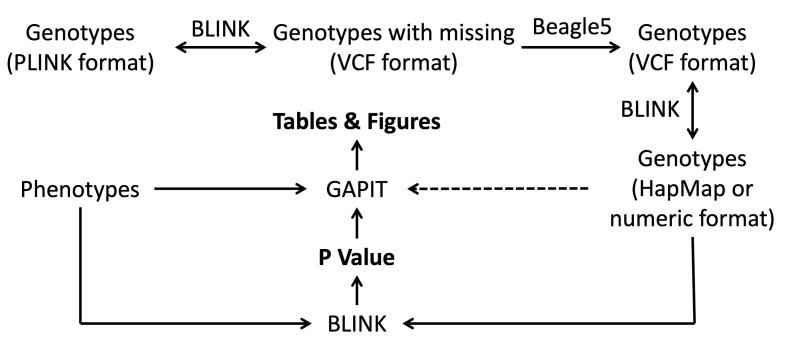
File format conversion, missing genotype imputation, and genome-wide association study. The path with a dashed line demands more memory than the paths with solid lines. The path with two arrows can be conducted in both directions.

We analyzed the rice data with only three software packages: BLINK for file format conversion and GWAS, BEAGLE for missing genotype imputation, and GAPIT for GWAS (Figure 1). BEAGLE was downloaded at https://faculty.washington.edu/browning/beagle/beagle.html for BEGEAL5. GAPIT was downloaded at https://zzlab.net/GAPIT, and BLINK was downloaded at https://zzlab.net/blink. GAPIT functions can be imported to the R library using a source command (source("http://raw.githubusercontent.com/jiabowang/GAPIT/refs/heads/master/gapit_functions.txt")).

1. Phenotypic outliers

Handling phenotypic outliers is a critical step in GWAS analysis to minimize false positives and ensure data reliability. Phenotypic outliers can lead to false positives when they randomly possess specific alleles for certain SNPs. Typically, extreme phenotypic values are removed from analyses to reduce false positives, as they constitute only a small proportion of data. For small samples, an alternative is to retain the outliers with their values pulled back to acceptable boundaries. In the case that a phenotype follows a normal distribution, outliers can be determined by the distances to the median. Three standard deviations from the median of phenotype values are commonly recommended as the upper boundary and lower boundary. The upper boundary is the median value plus three standard deviations. The lower boundary is the median value minus three standard deviations. Given the small size of the phenotype data file, both R and Excel are effective tools for identifying and removing outliers.

2. Genotype file format conversion

Ensuring the compatibility of genotype file formats is essential when working with different GWAS software packages, each of which has distinct requirements. Different software packages require different formats, especially for genotype files that exhibit a wide range of variation. For example, BEAGLE uses the VCF format for missing genotype imputation. GAPIT uses numerical or HapMap formats to import genotype files. Conversion of file formats is necessary for specific or multiple software packages. BLINK C version accepts genotype data in multiple formats, including BLINK text format, BLINK binary format, VCF format, HapMap format, and PLINK binary format. The BLINK C version can also output any of these formats. Therefore, the BLINK C version can be used to convert file formats. For example, rice genotype data, which contain missing genotypes, are saved in the PLINK format. We used the BLINK C version to convert to VCF format (Box 2), enabling its use with BEAGLE software for imputation.

**Box 2. Script for genotype file format conversion**

**Table:** 

map=read.table("HDRA-G6-4-SNP-MAP/HDRA-G6-4-final-snp-map.tsv",head=F) bim=read.table("HDRA-G6-4-RDP1-RDP2-NIAS/HDRA-G6-4-RDP1-RDP2-NIAS.bim",head=F) bim[,5:6]=map[,4:5] write.table(bim,"HDRA-G6-4-RDP1-RDP2-NIAS/HDRA-G6-4-RDP1-RDP2-NIAS.bim",quote=F,row.names = F,col.names = F) #convert PLINK format to VCF format system("./blink --file HDRA-G6-4-RDP1-RDP2-NIAS/HDRA-G6-4-RDP1-RDP2-NIAS --plink --compress --noimp --out rice") system("./blink --file rice --vcf --recode --out rice")

3. Missing genotype imputation

Proper management of missing values in phenotype, covariate, and genotype data is crucial for effective GWAS analysis across various software platforms. Missing values are not allowed by most software in phenotype and covariate data files. GAPIT and BLINK C version accept phenotype and covariate data files with missing values tagged “NaN”; however, samples with missing values are removed before the analysis. Genotype missing values are also not allowed in most software and should be imputed with sophisticated models before GWAS, especially if there is a substantial proportion of missing genotypes. GAPIT and BLINK C version accept missing values in a genotype data file and impute them using the major genotype method. For genotypes with a significant proportion of missing genotype data, we recommend using sophisticated imputation software, such as BEAGLE [44] (Box 3), for more robust results.

**Box 3. Script to impute missing genotypes**

**Table:** 

#run beagle5 to impute missing genotype. beagle5 could be downloaded from https://faculty.washington.edu/browning/beagle/beagle.html system("java -Xmx20000m -jar beagle.jar gt=rice.vcf out=test") #create txt file zlines <- readLines("HDRA-G6-4-RDP1-RDP2-NIAS/HDRA-G6-4-RDP1-RDP2-NIAS.fam") zline <- "FID IID father mother sex phenotype" zlines <- c(zline,zlines) writeLines(zlines,"rice.txt") system("./blink --file test --vcf --compress --out rice") system("./blink --file rice --hapmap --recode --out rice") system("awk '{print $2}' rice.txt > ID.txt") #load SNP data, map, and phenotype trait1=read.table("phenoAvLen_G6_4_RDP12_ALL/phenoAvLen_G6_4_RDP12_ALL.txt",head=T) #match and convert sample ID between SNP data and phenotype data ID=read.table("ID.txt",head=T) trait1[,1]=paste("IRGC",trait1[,1],sep='') trait=read.delim("HDRA-G6-4-RDP1-RDP2-NIAS/HDRA-G6-4-RDP1-RDP2-NIAS-sativa-only.sample_map.rev2.tsv",head=F) trait=merge(trait,ID,by.x="V2",by.y="IID") trait=merge(trait,trait1,by.x="V3",by.y="FID") myG=read.table("rice.hmp",head=F,check.names = F) myY=trait[,c(2,12)] #Run BLINK in GAPIT source("source("http://raw.githubusercontent.com/jiabowang/GAPIT/refs/heads/master/gapit_functions.txt") myGAPIT=GAPIT(Y=myY,G=myG,PCA.total=3,model="BLINK")

4. Minor allele frequency control

Managing rare variants is essential in GWAS to reduce the risk of false positives and ensure reliable results. Rare variants can also be a source of false positives. SNPs with low minor allele frequency (MAF) should be either removed before analysis or interpreted with caution if they are positive. We recommend removing at least SNPs with MAF < 0.05. This operation can be conducted in GAPIT using SNP.MAF = 0.05. The default value, SNP.MAF = 0, retains all SNPs without filtering.

5. Population structure analysis

Controlling population structure through principal components (PCs) is a key strategy in GWAS to improve the accuracy of association results. PCs are calculated using all genotype data and can be fitted as covariates to account for population structure. GAPIT users may set the number of top PCs through the flag PCA.total to add PCs in GWAS analysis (e.g., PCA.total=3 includes the top 3 PCs in the analysis). The selected PCs are fitted as covariates (fixed effects) to incorporate population structure. Alternatively, PCs can be precomputed using BLINK (--pca) as the external covariate file for GAPIT and the BLINK C version. For PC calculation, BLINK uses the PLINK binary format.

6. Modeling

Selecting an appropriate statistical model is fundamental in GWAS, and GAPIT offers a wide range of options to meet varying analytical needs. GAPIT supports multiple models, including GLM, MLM [5], CMLM [12], ECMLM [38], SUPER [39], MLMM [36], FarmCPU [45], and BLINK [26]. Among these, the last three (MLMM, FarmCPU, and BLINK) are multiple-locus models, while the others are single-locus models. Users can specify any model by using the model statement. For example, model = "FarmCPU" will specify FarmCPU model for the analysis. BLINK is set as the default due to its high statistical power and computing efficiency (Graphical overview). GAPIT also supports running GWAS with multiple models simultaneously. For example, the following statement conducts GWAS with both FarmCPU and BLINK using phenotype myY, genotype data myGD, and genotype map myGM with three principal components as covariates:

GAPIT(Y = myY, GD = myGD, GM = myGM, PCA.total = 3, model = c("FarmCPU","BLINK")).

7. BLINK for big data

Efficiently processing large genotype datasets is a significant challenge in GWAS, often requiring optimized computational tools to manage resource demands. For researchers dealing with large genotype datasets, conducting GWAS can be computationally demanding. In such cases, the computational cost of using the GAPIT package can be prohibitively high. As a solution, the BLINK C version has been developed to allow for efficient GWAS analysis with large datasets. Compared to the BLINK R version in GAPIT, the BLINK C version requires less memory and CPU time, making it an attractive option for researchers dealing with big data (Box 4). To facilitate data processing, we recommend converting the genotype data file to the BLINK binary format as the input file. Additionally, the BLINK C version can utilize multiple CPU threads or GPU threads to further accelerate GWAS, offering researchers flexible options to optimize their computational resources.

**Box 4. Script to run BLINK C version**

**Table:** 

# Calculate PCA and conduct GWAS using BLINK C version blink --file myData --plink --pca 3 --out test blink --file myData --plink --gwas --out test

**Result interpretation**

The Rice 3000 genome project dataset is an open, accessible, high-density SNP dataset for GWAS in rice. A total of 700,000 raw SNPs were obtained on 847 varieties genotyped by a high-density rice array. We imputed the missing genotypes using BEAGLE without a reference. With the imputed data, we summarized the properties of this genotype dataset using GAPIT, including heterozygosity, MAF, and LD across the rice genome (Figure S2). Linkage disequilibrium was calculated as the squared Pearson correlation coefficient between adjacent SNPs. We noticed a sparse area on chromosome 5 for MAF above 0.3. It is unclear what causes this phenomenon.

Heterozygosity was further summarized for individual samples (Figure S3a) and markers (Figure S3b). The distribution of MAF demonstrates that there were more markers with low and high MAF (Figure S3c), suggesting more new variants, genotyping errors, or selection.

LD was also displayed as the Pearson correlation between adjacent markers. There were more positive correlations than negative correlations (Figure S4d). Genotypes were coded as 0 and 2 for homozygous genotypes and 1 for heterozygous genotypes. The homozygous genotypes with a nucleotide in low alphabet order were assigned a value of 0, and those with a nucleotide in higher alphabet order were assigned a value of 2. This coding should not cause this phenomenon, and biological reasons remain unclear. Most markers are within 50 kb of their adjacent markers (Figure S4e). Linkage disequilibrium (LD) decay over distance is displayed as both R (Figure S4c) and R square (Figure S4f).

Principal component analyses (PCA) were also conducted in GAPIT using all SNPs. The first three PCs are illustrated pairwise and in 3D (Figure S5). The second PC differentiates the individuals with low PC1 values, and the third PC differentiates the individuals with high PC1 values. We noticed that none of the PCs explained the bimodal distribution of grain length.

Phenotypes are visualized as scatter plots, box plots, histograms, and density and accumulative density distributions (Figure S6). The scatter plots, histograms, and density plots intuitively demonstrate that rice grain length has at least a bimodal distribution (Figure S6a, c, and d). The box plot barely reveals this phenomenon. The bimodal distribution suggests that major genes may exist to control the phenotype.

Although an association was not found between the PCs and the bimodal distribution of grain length, there could be population stratification for the trait that may cause either false positives or reduction of power. Therefore, the first three PCs were used as the covariates in GWAS using multiple GWAS models, including BLINK, FarmCPU, and MLM implemented in GAPIT. The SNPs with MAF < 0.01 were filtered out using the flag SNP.MAF = 0.01. Associations were determined by a type I error of 5% genome-wide threshold after Bonferroni correction (0.05/700,000 = 7.14e-8).

**Figure 2.**
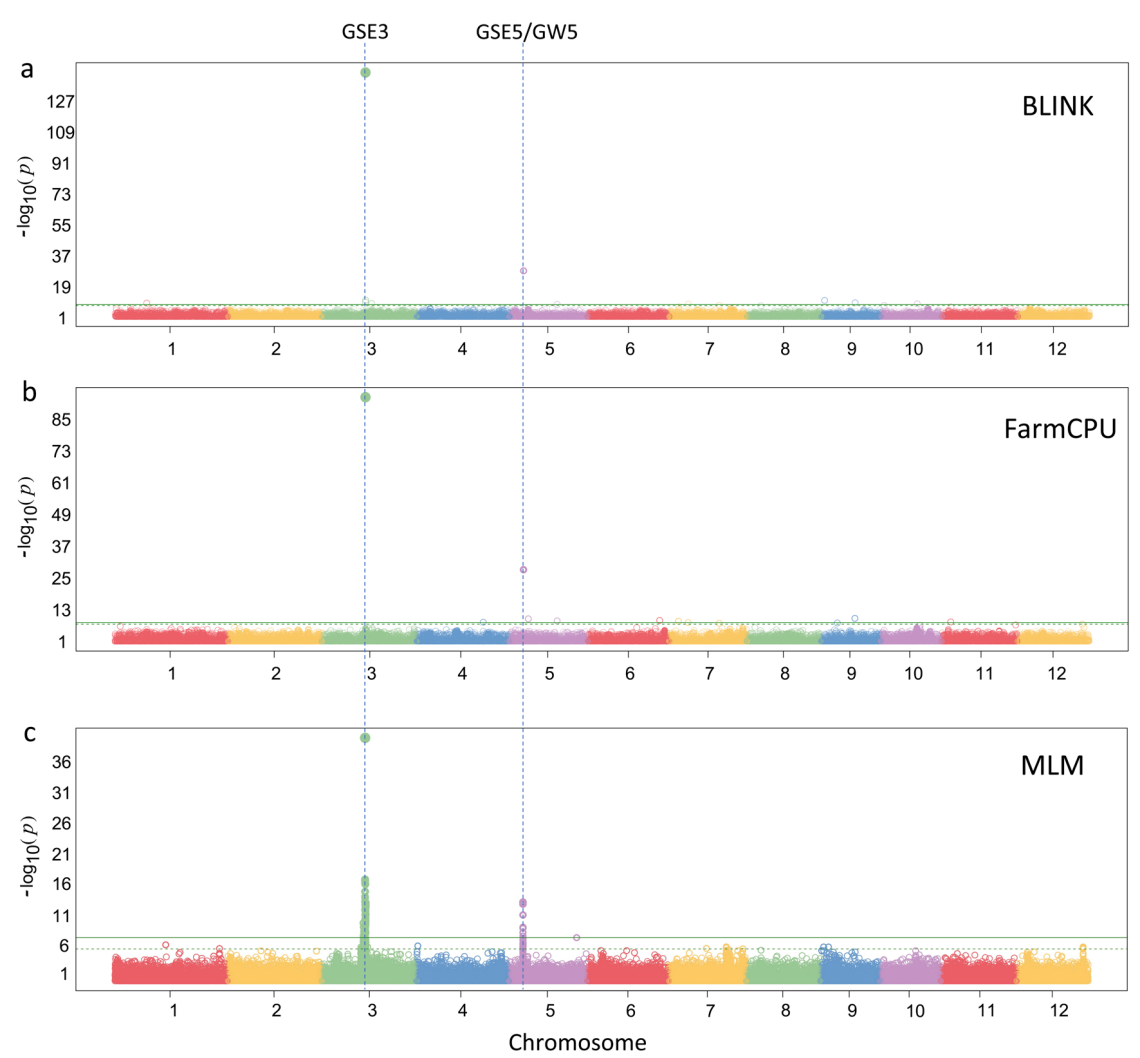
Manhattan plots for genome-wide association study (GWAS) results of rice grain length. GWAS was conducted with three models: BLINK (a), FarmCPU (b), and mixed linear model (MLM) (c).

There are six known genes for rice grain length (i.e., *GSE3, GSE5/GW5, qGL3, GL4, GL7/GW7*, and *TGW6*) [46–51]. All three models revealed the two known genes: *GSE3* and *GSE5/GW5*. BLINK detected ten SNPs, including four located close to these two known genes (Table 2). FarmCPU detected 9 SNPs, two of which were located near the same two known genes. MLM detected 191 SNPs, also located near these two known genes. The association signals from BLINK are stronger than those from FarmCPU and MLM (Figure 2).

Additionally, BLINK was the only model that could be finished on a laptop (32 GB memory), as the process of FarmCPU and MLM was terminated due to memory limitation. FarmCPU and MLM were successfully conducted on a server machine, which has 500 GB of memory (both FarmCPU and MLM need ~40G memory). For big data, the BLINK C version is recommended as it does not read the entire genotype data into memory at once. Genotype data are loaded in parts as they are examined in GWAS. A dataset with one million markers and half a million individuals can be processed on a laptop within a few hours, according to the performance reports on BLINK [26]. The computing time on the rice data with 847 lines and 0.7 M SNPs is less than three minutes using BLINK to conduct PCA and calculate P values and using GAPIT to plot P values. GAPIT took more than two hours to complete these three tasks (Table S2).

The results of the GWAS were visualized using GAPIT, yielding several informative plots. First, the PCA components plot (Figure S7a) was generated to illustrate the principal components analysis, highlighting the top three PCs. Second, the kinship tree and heatmap plot (Figure S7b) were produced to depict the genetic relatedness among individuals and the corresponding heatmap of genetic distances, providing insights into the population structure. Lastly, the pie plot on the genetic effects (Figure S7c) was created to summarize the proportion of genetic variance explained by polygenes across genome, offering a clear overview of the genetic contributions to the phenotypic traits under study. These visualizations collectively enhance the interpretation of the GWAS results, enabling a comprehensive understanding of the genetic architecture of the traits examined.

Several diagnostic plots were provided by GAPIT to evaluate the inflation control of observed P values against a null distribution, the relationship between effects and MAF, and the relationship between phenotypic variance explained and MAF for the associated SNPs (Figure S8). The control of P value inflation using BLINK is illustrated by the quantile-quantile plot. The majority of the SNPs (>99.99%) have P values that align with expectation. There are only a few SNPs with P values exceeding expectation. Their P values, estimated effects, phenotypic variance explained (PVE), and MAF are illustrated in Table 2.

**Table 2.** Profiles of significant SNPs from BLINK

SNP	CHR	Position	P value	MAF	Effect	PVE (%)	Gene	Distance
1.12197820	1	12,198,847	1.38E-08	0.35	-0.10	0.69	NA	-
3.16709886	3	16,711,241	3.34E-09	0.03	-0.20	6.03	NA	22K
3.16732086	3	16,733,441	1.02E-142	0.37	-0.47	11.47	GS3	0K
3.16786622	3	16,787,749	2.24E-10	0.11	0.14	3.57	NA	54K
3.19095745	3	19,096,986	2.37E-08	0.12	0.10	0.65	NA	-
5.5371749	5	5,371,772	2.28E-27	0.48	0.14	1.48	GSE5/GW5	5K
7.7243264	7	7,244,260	5.50E-08	0.5	0.07	0.93	NA	-
9.1311939	9	1,312,940	3.72E-10	0.02	-0.26	3.18	NA	-
9.12961389	9	12,962,391	9.25E-09	0.02	0.25	10.61	NA	-
10.13696722	10	13,767,954	3.85E-08	0.42	0.08	0.38	NA	-

**Figure 3.**
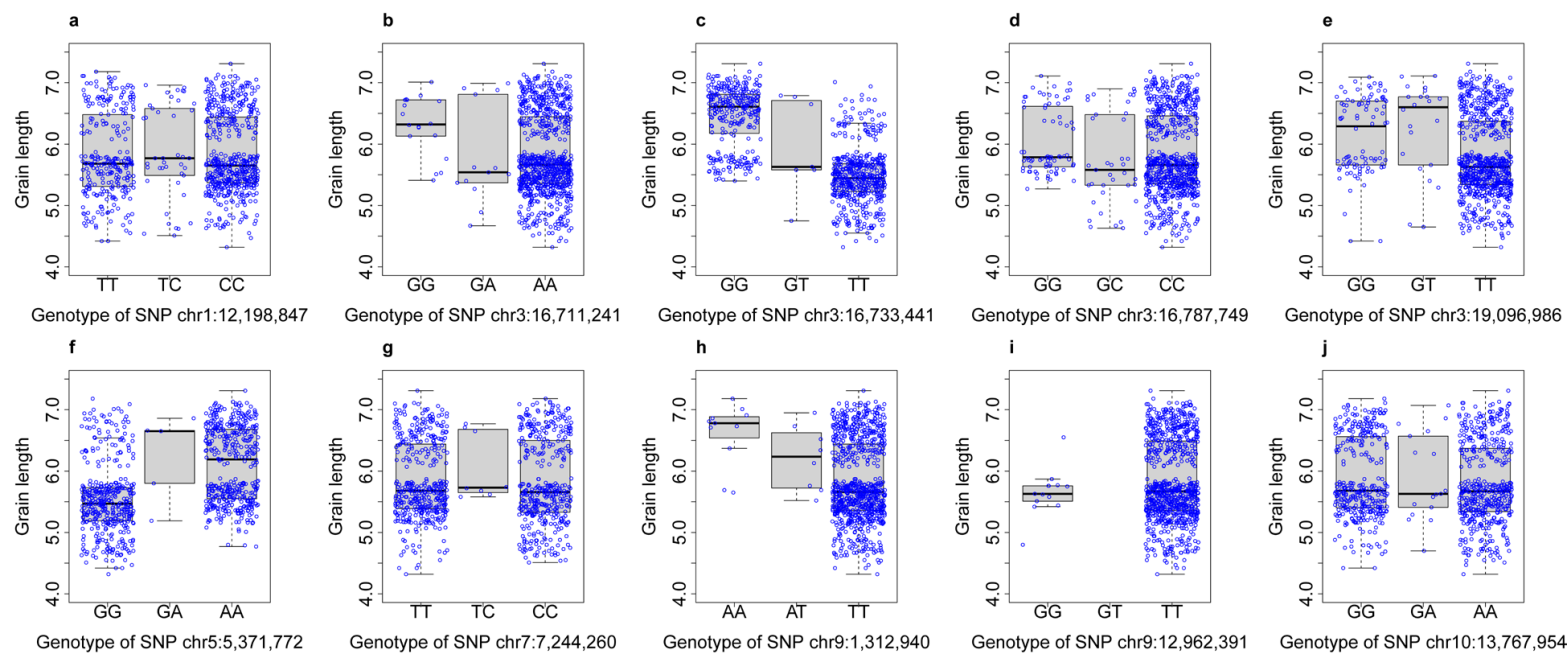
Distribution of grain length across genotypes of associated SNPs. Quantile boxplots display the distribution of grain length over the genotypes (GG, GT, and TT) of the most significant SNP on chromosome 3 at 16,733,441 bp (c). The genotypes of all ten associated SNPs are illustrated by the boxplots (a–j).

The GS3 gene on chromosome 3 is a major gene for grain length [52–55]. One of the SNPs on the gene (chr3:16733441) is the most significant SNP and explains the most phenotypic variation (11.47%). The average grain length of the two homozygous genotypes are one unit apart for the associated SNP (Figure 3), compared to the standard deviation of 0.48 in genotype GG and 0.45 in genotype TT. This SNP substantially explains the bimodal distribution of grain length. Most individuals with GG have a grain length lower than 6, and most individuals with TT have a grain length higher than 6. However, there are still two clusters wihtin the genotypes of the most significant SNP. The remaining associated SNPs partially explain the two clusters.

**Discussion and conclusion**

GAPIT provides a variety of GWAS models, including the BLINK model, which is also implemented in the BLINK C version. Beyond GWAS, GAPIT offers additional functions for input data quality control and result interpretation, such as imputing missing genotypes using the major genotype method. However, caution is advised when using GAPIT’s imputation function, particularly for datasets with a substantial proportion of missing genotypes. In such cases, advanced imputation software like BEAGLE is recommended for preprocessing before conducting GWAS with GAPIT or BLINK. While GAPIT is convenient for checking input data, performing GWAS with multiple models, and generating user-friendly output for interpretation, its capacity is significantly limited when handling large datasets compared to the BLINK C version. For big data, the BLINK C version provides a practical solution for efficiently calculating P values. Subsequently, GAPIT can be employed to visualize these P values from BLINK, enhancing the interpretation of results.

In this study, we present a comprehensive protocol for conducting GWAS using the BEAGLE, BLINK, and GAPIT packages. We outline a step-by-step process for data format conversion, GWAS analysis, and result interpretation. We evaluate the performance of multiple GWAS models within GAPIT, demonstrating that the BLINK model achieves the highest statistical power. To enhance the presentation of GWAS findings, we generate various plots using GAPIT’s visualization tools. Our results emphasize the effectiveness of GAPIT, particularly with the BLINK model, in streamlining GWAS analysis. GAPIT’s visualization tools provide clear and concise representations of results, facilitating easy interpretation for researchers. Additionally, the BLINK C version offers an optimized solution for large-scale GWAS analysis. This protocol serves as a valuable guide for researchers seeking to utilize these tools in GWAS studies.

## Validation of protocol

This protocol (or parts of it) has been used and validated in the following research articles using GAPIT [56,57], BLINK in GAPIT [58,59,60], BLINK C version [56,61,62], and BEAGLE [63,64,65]:

• Horvath et al. [56]. Genome-Wide Association Studies and Transcriptome Changes during Acclimation and Deacclimation in Divergent Brassica napus Varieties. *Int J Mol Sci.* 21(23): 9148. https://doi.org/10.3390/ijms21239148

• Ingole et al. [57]. Genome-wide association analysis for pollen viability under heat stress in peanut. *Plant Stress.* 15: 100760. https://doi.org/10.1016/j.stress.2025.100760

• Bertolini et al. [58]. Regulatory variation controlling architectural pleiotropy in maize. *Nat Commun.* 16(1): 2140. https://doi.org/10.1038/s41467-025-56884-w

• Nyine et al. [59]. Genomic signals of ecogeographic adaptation in a wild relative are associated with improved wheat performance under drought stress. *Genome Biol.* 26(1): 35. https://doi.org/10.1186/s13059-025-03500-1

• Trubanová et al. [60]. Genome-specific association study (GSAS) for exploration of variability in hemp (Cannabis sativa). *Sci Rep.* 15(1): 8371. https://doi.org/10.1038/s41598-025-92168-5

• Zhao et al. [61]. Trait associations in the pangenome of pigeon pea (*Cajanus cajan). Plant Biotechnol J.* 18(9): 1946–1954. https://doi.org/10.1111/pbi.13354

• Raiyemo et al. [62]. Chromosome‐level assemblies of *Amaranthus palmeri, Amaranthus retroflexus*, and *Amaranthus hybridus* allow for genomic comparisons and identification of a sex‐determining region. *Plant J.* 121(4): e70027. https://doi.org/10.1111/tpj.70027

• Kassie et al. [63]. Genome-wide association analysis of Septoria tritici blotch for adult plant resistance in elite bread wheat (*Triticum aestivum* L) genotypes. *PLoS One.* 20(3): e0317603. https://doi.org/10.1371/journal.pone.0317603

• Križanac et al. [64]. Sequence-based GWAS in 180,000 German Holstein cattle reveals new candidate variants for milk production traits. *Genet Sel Evol.* 57(1): 3. https://doi.org/10.1186/s12711-025-00951-9

• Zhu et al. [65]. Multiple strategies association revealed functional candidate FASN gene for fatty acid composition in cattle. *Commun Biol.* 8(1): e1038/s42003–025–07604–z. https://doi.org/10.1038/s42003-025-07604-z

• Shen et al. [66]. The weak association between hypoxia tolerance and thermal tolerance increases the susceptibility of abalone to climate change. *Environ Res.* 264: 120324. https://doi.org/10.1016/j.envres.2024.120324

• Zhang et al. [67]. The amphipod genome reveals population dynamics and adaptations to hadal environment. *Cell.* 188(5): 1378–1392.e18. https://doi.org/10.1016/j.cell.2025.01.030

• Zhang et al. [68]. SPDC‐HG: An accelerator of genomic hybrid breeding in maize. *Plant Biotechnol J.* e70011. https://doi.org/10.1111/pbi.70011

## Supplementary information

The following supporting information can be downloaded here:

1. Table S1. Abbreviation description

2. Table S2. Computing time of using different software at different phases of GWAS

3. Figure S1. Joint Manhattan plot with multiple models in GAPIT

4. Figure S2. Heterozygosity, minor allele frequency, and linkage disequilibrium across the rice genome

5. Figure S3. Heterozygosity and minor allele frequency of single nucleotide polymorphisms (SNPs) in rice data

6. Figure S4. Marker density and linkage disequilibrium across rice genome and their relationship

7. Figure S5. Principal component analysis of SNP data on different scales

8. Figure S6. Phenotype overview of rice grain length

9. Figure S7. The gallery of GWAS results illustrated by GAPIT

10. Figure S8. Diagnoses of associations against null distribution and minor allele frequency

All code used for this paper is available at https://github.com/MingLiang-Li/GWAS_protocol.
